# LightDenseYOLO: A Fast and Accurate Marker Tracker for Autonomous UAV Landing by Visible Light Camera Sensor on Drone

**DOI:** 10.3390/s18061703

**Published:** 2018-05-24

**Authors:** Phong Ha Nguyen, Muhammad Arsalan, Ja Hyung Koo, Rizwan Ali Naqvi, Noi Quang Truong, Kang Ryoung Park

**Affiliations:** Division of Electronics and Electrical Engineering, Dongguk University, 30 Pildong-ro 1-gil, Jung-gu, Seoul 100-715, Korea; stormwindvn@dongguk.edu (P.H.N.); arsal@dongguk.edu (M.A.); koo6190@naver.com (J.H.K.); rizwanali@dongguk.edu (R.A.N.); noitq@dongguk.edu (N.Q.T.)

**Keywords:** unmanned aerial vehicle, autonomous landing, real-time marker detection, lightDenseYOLO, visible light camera sensor on drone

## Abstract

Autonomous landing of an unmanned aerial vehicle or a drone is a challenging problem for the robotics research community. Previous researchers have attempted to solve this problem by combining multiple sensors such as global positioning system (GPS) receivers, inertial measurement unit, and multiple camera systems. Although these approaches successfully estimate an unmanned aerial vehicle location during landing, many calibration processes are required to achieve good detection accuracy. In addition, cases where drones operate in heterogeneous areas with no GPS signal should be considered. To overcome these problems, we determined how to safely land a drone in a GPS-denied environment using our remote-marker-based tracking algorithm based on a single visible-light-camera sensor. Instead of using hand-crafted features, our algorithm includes a convolutional neural network named lightDenseYOLO to extract trained features from an input image to predict a marker’s location by visible light camera sensor on drone. Experimental results show that our method significantly outperforms state-of-the-art object trackers both using and not using convolutional neural network in terms of both accuracy and processing time.

## 1. Introduction

The evolution of the self-driving vehicle is about to shift into overdrive thanks to the recent advancements of computer vision and artificial intelligence. Companies such as Tesla, Google, Intel and Amazon are spending billions of dollars to develop cutting-edge autonomous systems about to transform how we think about transportation in the next decade. Autonomous unmanned aerial vehicles (UAVs) and cars are receiving the most attention because their practicality in daily life. UAVs have proved their usefulness in a variety of areas, especially for military purposes such as search and rescue, monitoring, surveillance, as well as applications such as 3D reconstruction, transportation, and artistic photography. However, most drones or UAVs are not truly autonomous and are operated remotely by a human controller from the ground. Therefore, the next generation drone requires a self-controlling function to fly in an unstructured environment and perform autonomous landing. Recently, logistics company Matternet has announced a permanent autonomous drone network that would fly lab samples like blood tests and other diagnostics between hospital facilities, clinics, and labs [1]. Amazon Prime Air is the first drone delivery technology to receive online orders and ship packages to customers within 30 min [2] but they have yet to determine how to deal with safety issues such as controlling drones to navigate over GPS-denied regions and landing at precise locations.

Our previous work attempted to solve the problem of autonomous drone landing by using hand-crafted features extracted by a remote-marker-based tracking algorithm using a single visible light camera, and we successfully tracked a marker at a height of 10 m [3]. However, it is difficult to be apply when a drone is higher than 10 m because of the low resolution of the marker in the drone camera image which is often the case with drone landing.

Many researchers are using deep-learning techniques to extract trained deep features [4]. They have exhibited magnificent performance in many challenging tasks that traditionally depend on hand-crafted features such as localization [5], monitoring [6,7], classification [8], human crowd detection [9], self-stabilization [10,11], obstacle and crash avoidance [12,13,14], perception of forest or mountain trails [15,16] and object tracking [17,18]. In this research, we introduce an enhanced method for remote-marker-based tracking to exploit the trained features extracted from a proposed convolutional neural network (CNN) named lightDenseYOLO and perform marker tracking at very long distances, the furthest being 50 m. We also introduce a post-processing Profile Checker v2 algorithm to predict marker direction and refine the predicted center made by lightDenseYOLO. Therefore, we can track the location of a marker during the landing operation in a GPS denied environment at a very long distance.

The outline of the paper is organized as follows. In Section 2, we present recent works related to autonomous landing methods for drones. In Section 3, we introduce our approach on visual marker tracking algorithm with lightDenseYOLO. In Section 4, we compare tracking performances in various environments including both the desktop computer and the Snapdragon 835 mobile hardware development kit [19]. We conclude our research in Section 5.

## 2. Related Works

Previous research on vision-based autonomous drone landing can be categorized into passive and active methods. Previous studies on passive methods have taken advantage of multiple camera sensors distributed on the ground and sophisticated set ups for both the drone and ground environments were required. Zhou et al. [20] attempted to solve the autonomous unmanned aerial vehicle (UAV) landing problem by proposing a ground-based multisensory fusion system including a pan-tilt unit (PTU), infrared camera, and distance-sensor based on ultra-wideband radar. Although this method showed promise, the multisensory system requires a meticulous time-consuming calibration process. A stereo ground vision-based system including two pan-tilt units was proposed by Tang et al. [21]. The aircraft position was detected by the Chan-Vese model-based object-detection algorithm [22] and then updated by an extended Kalman filter [23] to enhance the localization accuracy. Recent work by Yang et al. [24] showed accurate UAV landing performance in a GPS denied-environment using a ground-based near infrared camera system. The ground system successfully tracked an infrared laser lamp mounted on the nose of the UAV from a distance of 1 km. Although the above research has shown remarkable tracking performance in urban areas at low altitude operations, setting up a ground station with complicated equipment was still a problem.

Research on active methods has overcome these difficulties by using an on-board camera mounted on a UAV to detect a safe region or marker on the ground to perform accurate landing. They can be classified into marker-less-based and marker-based approaches. With respect to the former category, Forster et al. proposed a method to capture images from a downward-facing camera to generate a 3D terrain depth map, a secure area for landing was located by a landing spot detection algorithm [25]. While this method worked well in both indoor and outdoor scenarios, the depth estimation algorithm was only tested at a low altitude of 5 m and was time-consuming. Gui et al. [26] proposed an algorithm to estimate the relative position of a UAV and a runway to perform autonomous landing based on the images captured from an infrared camera. Although this method successfully worked at both day time and night time, it requires a complicated set-up of multiple infrared lamps on the runway, making it impractical in narrow spaces.

To overcome the limitation of the marker-less approaches, several marker-based methods have been researched. The marker center and direction are predicted from each input image to guide the drone to land at the marker central position with the correct orientation by utilizing extracted hand-crafted features. Lin et al. [27] used a single downward-facing visible-light camera to track the position of an H-pattern landing target for the autonomous landing operation. The line segments were detected from binarized inputs to find the potential clustering of points containing the target. A hexagon marker with a black background and several concentric white rings was proposed by Lange et al. [28] to easily find the radius in pixel of the rings by the contour detection algorithm. The distance between the target and UAV was estimated by the actual (known) radius in 3D space and detected one in image of these rings based on intrinsic camera parameters. 

Recently, many studies [29,30] used a fiducial marker such as an April Tag [31] as a landing target to perform autonomous drone landing on a moving platform. While the fiducial April Tag marker is robust to be detected in many difficult challenges such as low image resolution, severe rotation, heavy occlusions and light variations, the detection range was limited to approximately 7 m [32]. Although these studies successfully tracked the marker during the day, they would fail if the experiment were to be conducted at night. In previous research [33], they also proposed the method for autonomous landing of multirotor UAV based on the moving pad including April Tag marker. The fusion of inertial measurement with the estimated pose was adopted to ensure a high sampling rate, and to increase the maneuverability of the vehicle. Two filters were designed to perform the fusion, an extended H_∞_ (EH_∞_) and an extended Kalman filter (EKF). Although their accuracies were very high, their method requires the information of inertial measurement. In addition, their experiments were done only in indoor environments, and the height between the UAV and marker was limited (less than 5 m) in real world experiment due to the characteristics of April Tag marker used in their method [33]. Therefore, their method cannot be compared with our method of marker detection which is operated in outdoor and at very long distance (50 m) between the UAV and marker.

Other researchers used a thermal imager to identify the marker for the night scenario [34,35]. The landing target actively emits infrared light to enhance the detection accuracy under low-light conditions. However, most of the conventional drone systems include only a visible-light camera; therefore, a drone carrying a thermal camera sensor was a compulsory requirement for these studies. Viewing the problem from a different aspect, Polvara et al. exploited deep-trained features extracted from a hierarchy of double-deep Q-networks [36]. This network acted like a high-level control policy for navigating the UAV toward the marker in an indoor environment. However, there was a gap between the indoor and outdoor environments.

To overcome previous researches limitations, we propose a long distance remote-marker-based tracking algorithm based on lightDenseYOLO using a single visible-light camera sensor. We also show that our algorithm can be operated with the commercially available Snapdragon 835 mobile hardware development kit [19]. Using this kit, we managed to operate our marker detection algorithm in real-time. Our research is novel compared to previous works in the following three ways:

(1) Our method uses a lightweight CNN named lightDenseYOLO to perform an initial prediction of the marker location and then refine the predicted results with a new Profile Checker v2 algorithm. By doing so, our method can detect and track a marker from 50 m.
(2) The proposed lightDenseYOLO maintains a balance between speed and accuracy. Our approach has a similar detection performance with state-of-the-art faster region-based CNN (R-CNN) [37] and executes five times faster. All experiments were tested on both a desktop computer and the Snapdragon 835 mobile hardware development kit [19].
(3) Our new dataset includes images taken from both long and close distances, and we made our dataset, trained models of lightDenseYOLO and algorithms available to the public for other researchers to compare and evaluate its performance [38].

A comparison of previous methods employed for autonomous drone landing with our method is summarized in Table 1.

## 3. Proposed Method

### 3.1. Long-Distance Marker-Based Tracking Algorithm

The size of our marker is 1 m ×1 m [3]. In this study, we solve the problem of autonomous drone landing by proposing a long-distance marker-based tracking algorithm using the same marker. There are two major differences between our current research algorithm and our earlier one [3]. First, we replaced the adaptive template matching (ATM) algorithm in [3] with our proposed CNN lightDenseYOLO marker detector. Our lightDenseYOLO is the combination of two components: lightDenseNet as the CNN feature extractor and YOLO v2 [39] as the marker detection module. Second, we introduced Profile Checker v2 to further enhance the predicted results.

**Figure 1:**
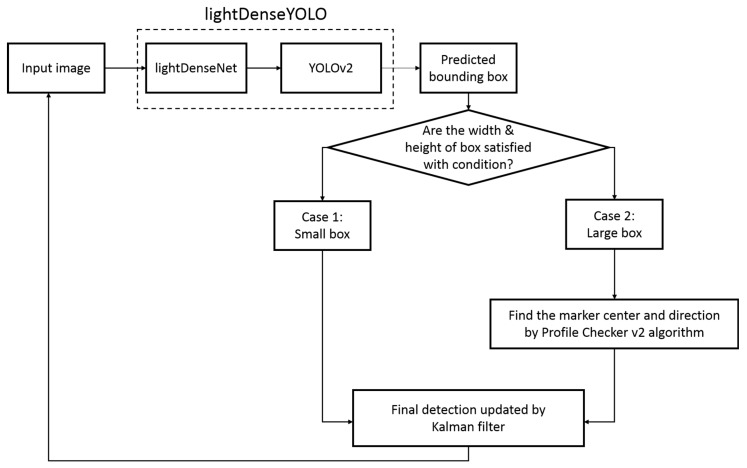
Flowchart of proposed far distance marker-based tracking algorithm.

Figure 1 shows the overall flowchart of our proposed method. We resized the original input image taken by drone camera from 1280 × 720 pixel (px) to 320 × 320 px to fit the input of our CNN lightDenseYOLO. The output of lightDenseYOLO was a set of bounding boxes which potentially include a marker. Among these predicted boxes, we selected the box with the highest confidence score. However, when the distance between the drone and the marker was large, the predicted box was very small, and it was difficult to predict the direction of the marker inside it. Therefore, we categorized the predicted bounding box based on its size. In our research, a box with width and height larger than 150 px was a “large” box and a “small box” had a width or height smaller than 150 px. If the predicted box was a small box, the center of the predicted box was chosen to be the marker center. If a large box was detected, we extracted the predicted box as an image and put it into the Profile Checker v2 algorithm to find a more accurate marker center and direction. We also used a Kalman filter [40] to increase the accuracy of the obtained center. Our previous study showed that using a Kalman filter not only helps to stabilize the overall prediction but also helps deal with occlusion and blurring.

Next, we describe the proposed lightDenseYOLO network for marker detection followed by the Profile Checker v2 algorithm.

### 3.2. Marker Detection with lightDenseYOLO

We define our marker detection problem as general object detection with two classes of marker and background. Much research has been performed in recent years on object detection using CNNs. The state-of-the-art CNN object detector is Faster R-CNN which is based on a two-stage proposal-driven mechanism. The first stage generates possible candidate object locations by a region proposal network (RPN) and the second stage classifies each candidate location into one of several classes. Faster R-CNN achieves high accuracy on many challenging datasets, but the processing time is a major concern. It has been reported that it takes 200 ms (five frames per second (fps)) to process each image using a modern graphic card [37]. It is obvious that Faster R-CNN is not the optimal solution for real-time marker detection using an on-board processor. The one-stage detector YOLO (“You Only Look Once”) [41] and the upgraded version YOLO v2 [39] not only demonstrate promising results but also yield about 10 times faster detection speed with accuracy degradation within about 10% relative to Faster R-CNN.

Therefore, we propose a one-stage detector named lightDenseYOLO which combines lightDenseNet—a simplified version of densely connected convolutional networks (DenseNet) [42]—as the feature extractor and YOLO v2 (bounding box) predictor. Consequently, our new model not only inherits the good extraction features from DenseNet but also the robust object-detection speed of YOLO v2.

#### 3.2.1. LightDenseNet Architecture

Assuming a single image $x_{0}$ passing through a CNN with *L* layers, $x_{l}$ is the produced output of the *l*th layer. Each layer includes a non-linear transformation $H_{l}\left(\cdot \right)$ to obtain output feature maps. In particular, $H_{l}\left(\cdot \right)$ can be a composite function of operators such as convolution (conv), pooling, batch normalization (BN), or rectified linear unit (ReLU). Traditional feed-forward networks such as AlexNet [43], visual geometry group (VGG) [44] connect the output of the (*l* − 1)th layer as the input of the *l*th layer. We demonstrate such networks in [42] as

(1) $$x_{l}=H_{l}\left(x_{l-1}\right)$$

Residual network (ResNet) introduces a skip connection that expands the non-linear transformation with an identity shortcut [45] and is expressed as

(2) $$x_{l}=H_{l}\left(x_{l-1}\right)+x_{l-1}$$

In contrast to ResNet, the feature maps in DenseNet are not combined through summation before they are passed to the next layer. Instead, in each dense block, the feature maps of the current layer are concatenated with feature maps from previous layers as an additional channel. Therefore, the *l*th layer has *l* inputs which are feature maps from all preceding layers $x_{0},\ldots ,\;x_{l-1}$. Equation (3) shows how DenseNet performs a non-linear transformation at the *l*th layer [42]:

(3) $$x_{l}=H_{l}\left(\left[x_{0},x_{1},\ldots ,x_{l-1}\right]\right)$$

**Figure 2:**
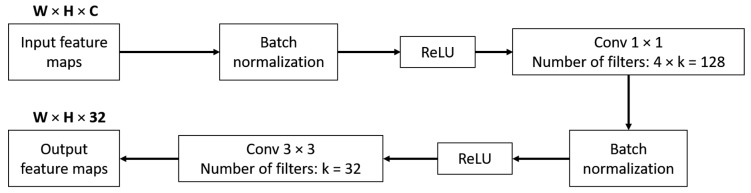
Bottleneck layer design.

In Equation (3), $\left[x_{0},x_{1},\ldots ,x_{l-1}\right]$ is the concatenation of feature maps produced from layer 0 to layer *l* − 1. Therefore, if the network is very deep, the number of channels of this concatenated feature maps is very large. To avoid making the network too large, DenseNet introduced a bottleneck layer to make sure that the number of output channels at each layer was a fixed number. Here, $H_{l}$ can be defined as a bottleneck layer which includes layers: BN, ReLU, 1 × 1 conv, BN, ReLU, and 3 × 3 conv. As shown in Figure 2, the bottleneck layer reduces the number of output channels from C to 32, where C is an arbitrary number larger than 32. In this research, we designed the number of channels of output feature maps at each bottleneck layer as 32.

**Figure 3:**
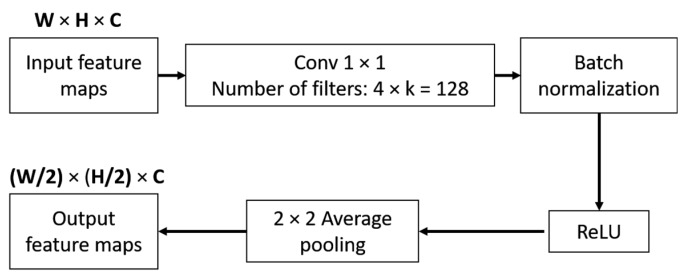
Transition layer design.

**Figure 4:**
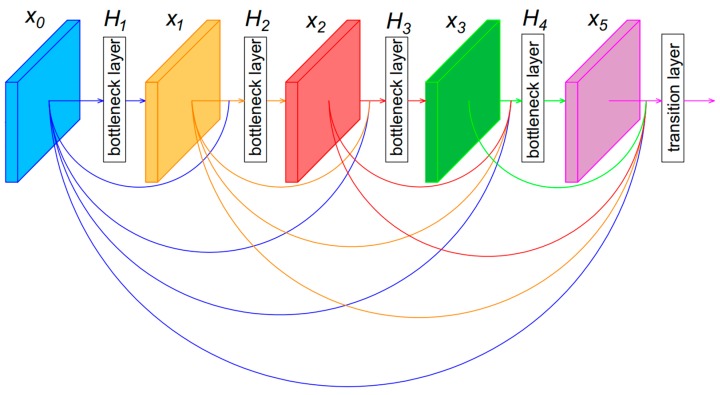
Dense block example.

An essential part of CNN is reducing the spatial size of feature maps by pooling. DenseNet divides a network into multiple dense blocks by transition layer. We can also define $H_{l}$ as a transition layer that includes BN, 1 × 1 conv and 2 × 2 average pooling. Figure 3 shows how a transition layer reduced the spatial size of the input feature maps by half. The output of the transition layer is the input to the next dense block. Figure 4 shows an example of a dense block with four bottleneck layers and a transition layer.

**Figure 5:**
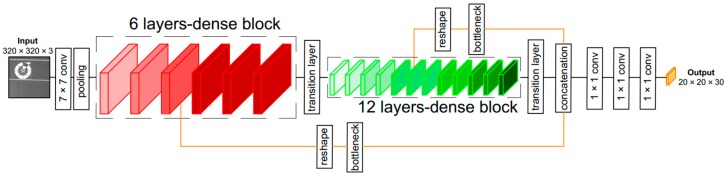
Proposed lightDenseNet architecture with two dense blocks and two lateral connections.

Figure 5 shows the proposed lightDenseNet architecture with two dense blocks along with two lateral connections. The previous DenseNet architecture with four dense blocks shows superior classification performance on the large ImageNet dataset, which includes more than one million images (for training and validation) and one thousand classes [42]. However, our marker dataset is much smaller than ImageNet and has only two classes of marker and background; therefore, we do not need a deep CNN architecture to extract features. Thus, a network with two dense blocks is a suitable solution. Moreover, a network with four dense blocks is too deep and increases the processing time; therefore, a two dense-block model is the optimal solution to balance between accuracy and processing speed.

To concatenate the higher- and lower-resolution features, we added two lateral connections to connect the middle layer of each dense block with the last output of the last transition layer. Each lateral connection includes a reshape layer and a bottleneck layer. First, the reshape layer changes the dimension of its input without changing its data. For example, if the dimensions of the middle layer of a dense block are W (width) and H (height) with C channels, the output dimensions after reshaping by a factor m are W/m, H/m, and C × m × m, respectively [39]. However, the number of channels (C × m × m) of this reshaped layer can be very large. Therefore, a bottleneck layer is utilized to reduce the number of channels after reshaping without changing the spatial size of the feature maps. In our research, we concatenated two produced outputs from two lateral connections with an output of the second transition layer into a single tensor. Moreover, three more convolution layers are added in lightDenseNet to match the desired output of the YOLO v2 detection algorithm.

#### 3.2.2. Comparisons on YOLO and YOLO v2 object detector

**Figure 6:**
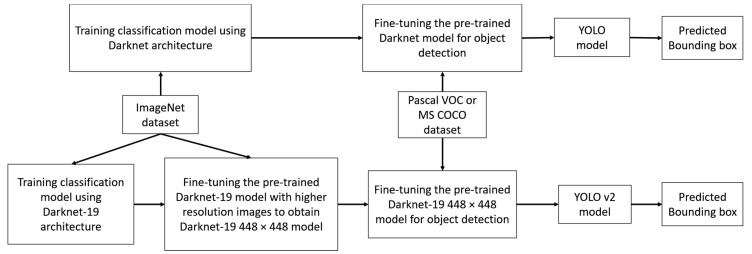
Overall flowchart of the training of YOLO and YOLO v2 for object detection

Our marker detection algorithm was based on the upgraded version of YOLO [41], named YOLO v2 [39]. Both YOLO and YOLO v2 require a pre-trained classification model as a backbone CNN architecture for the object detection task as shown in Figure 6.

There are differences between the backbone architectures of YOLO and YOLO v2. First, YOLO trains a classification CNN model called Darknet which includes 20 convolution layers, four max pooling layers, and a fully-connected layer using ImageNet dataset with an input size of 224 × 224 px. Learning to detect objects is the next step when YOLO fine-tunes the pre-trained Darknet model using higher 448 × 448-px resolution images from Pascal visual object classes (VOC) dataset. This is a difficult optimization problem because the network must perform two functions at the same time: learn to detect objects and adapt to the higher resolution input image. YOLO v2 solves this by proposing a new CNN architecture called Darknet-19 which has 19 convolution layers, five max pooling layers, and a fully-connected layer. Darknet-19 was initially trained on ImageNet dataset with 224 × 224 px input images for 160 epochs, and it was fine-tuned for 10 further epochs with 448 × 448 px input images. The final classification model of YOLO v2 is called Darknet-19 448 × 448.

The Darknet-19 448 × 448 model has better top-1 and top-5 accuracies on the standard ImageNet 1000 class classification than the Darknet-19 model and the original Darknet model [39]. In addition, the Darknet-19 448 × 448 model ensures there is no size mismatching between the input image of the training-from-scratch model and the fine-tuning model as shown in Table 2.

Both YOLO and YOLO v2 approach the object-detection task as a tensor-regression problem. The output tensors of YOLO and YOLO v2 include the information of predicted bounding boxes such as center coordinates, width, height, confidence score, and class probabilities. Passing an input image through a pre-trained model, both YOLO and YOLO v2 obtain a set of smaller-sized S×S feature maps and then divide that image into a grid with S×S grid cells. Each grid cell is responsible for predicting *B* bounding boxes. However, there is a difference on how YOLO and YOLO v2 find the optimal bounding boxes as the final predictions [39,41].

During fine-tuning for the object detection task, YOLO replaces the last fully-connected layer which was used for classification task with four convolution layers and two fully-connected layers. The output of YOLO last’s fully-connected layer is a S×S×(B×5+C) tensor, where *C* is the class probability and *B* is the number of predicted boxes. Each YOLO predicted box includes five values such as center coordinates (x, y), width, height, and a confidence score showing whether there is an object in the box or not. Bounding box width, height, and center coordinates are normalized to have values between 0 and 1. The final prediction score of one bounding box for each class is the multiplication of the confidence score and the class probability. Based on all the prediction scores of many predicted bounding boxes and a threshold value, a set of bounding boxes, which may contain an object, are acquired. However, there may be some overlapping bounding boxes among all detected boxes. Therefore, a non-maximum suppression algorithm is used to remove these redundant boxes [41].

**Figure 7:**
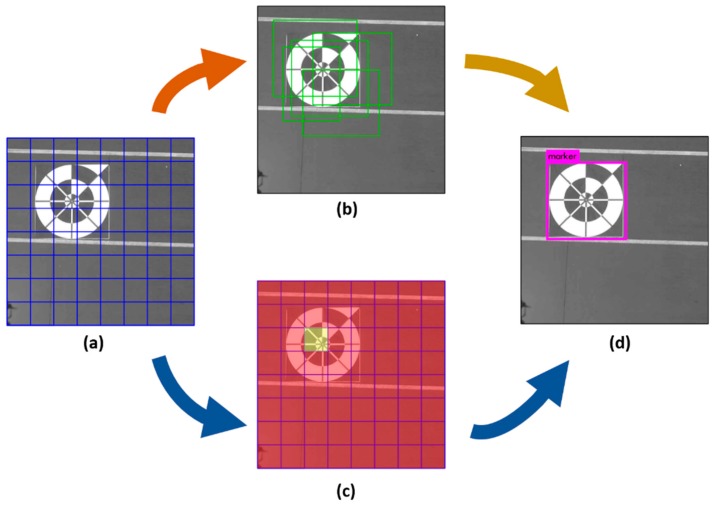
Example of YOLO v2 marker detection. (**a**) YOLO v2 divides the input image into S×S grid, and (**b**) each grid cell predicts five bounding boxes based on five prior anchor boxes. The predictions are stored in an S×S×(5×(5+C)) output tensor. This example shows how YOLO v2 predicts marker from an input image with a grid size *S* = 8 and *C* = 1 (number of classes to be detected) so that the size of the final output tensor is 8×8×30; (**c**) The yellow shaded cell shows higher potential of detecting marker compared to other red shaded cells; (**d**) The result of the detected marker.

YOLO v2 uses a different method than Faster R-CNN to predict bounding boxes based on prior anchor boxes. YOLO v2 anchor boxes are not chosen by hand but are determined by analyzing all the ground-truth bounding boxes in the training set. In our research, we trained the CNN of lightDenseYOLO using the same five prior anchor boxes used to train YOLO v2. Figure 7 shows an example of how YOLO v2 detects a marker by dividing the input into 8×8 grid cells (Figure 7a). Each grid cell is responsible for generating five bounding boxes based on five prior anchor boxes as shown in Figure 7b. If the center of the object from the ground-truth data is located inside the yellow cell in Figure 7c, that grid cell can contribute more than the red cells during training. Therefore, a single input image needs to pass through the neural network only one time during training and testing and enables a real-time processing speed (Figure 7d). Each YOLO v2 predicted box not only includes center coordinates, width, height, and confidence score but also the *C* class probability. Therefore, the size of the YOLO v2 final output tensor is S×S×(B×(5+C)) where *S* and *B* are the numbers of grids and bounding boxes, respectively. After the model is trained, the problem of selecting the best boxes becomes easier. In our research, we used *S*, *B*, and *C* as 20, 5, and 1, respectively, for testing of our lightDenseYOLO. If any bounding box had a class probability value larger than the threshold value, that box was deemed to have a higher potential to contain an object. Once again, the non-maximum suppression algorithm was used to determine the final detected boxes [39].

At each position of sliding-window, multiple candidate bounding boxes are simultaneously predicted based on the predetermined number of anchor boxes [37,39]. Each anchor box has various (predetermined) size and ratio of width to height to detect the objects having the various size and ratio. In our research, we use 5 anchor boxes. Based on each cell of S×S, five (predetermined) anchor boxes are used to predict five candidate bounding boxes. As shown in the following Equations (4)~(6), YOLO v2 predicts 5 bounding boxes at each cell in the output feature map. It predicts 5 coordinates for each bounding box, *t_x_*, *t_y_*, *t_w_*, *t_h_*, and *t_o_*. Here, *t_x_*, *t_y_*, *t_w_*, *t_h_*, and *t_o_* are the center x, center y, the width of box, height of box, and predicted probability, respectively. In case that the cell is offset from the top left corner of the image by (*b_x_*; *b_y_*) and the anchor box has width and height of *p_w_*, *p_h_*, the final coordinates of the predicted bounding box are *a_x_*, *a_y_*, *a_w_*, and *a_h_*as shown in Equations (4)~(6) [39]. Here, *sigm*(·), e(·), *Pr*(·), and *IOU*(·) show sigmoid activation function, exponential function, probability, and intersection over union, respectively.

*a_x_* = *sigm*(*t_x_*) + *b_x_, a_y_* = *sigm*(*t_y_*) + *b_y_* (4)

*a_w_*= *p_w_*e*^tw^*, *a_h_*= *p_h_*e*^th^* (5)

*Pr*(object) × *IOU*(*a*, *object*) = sigm(*t_o_*) (6)

In Figure 7c, the red shaded cell represent the predetermined cells of S×S, and the yellow shaded cell shows that having the high probability for including the marker center among the cells of S×S.

In addition, YOLO v2 removes all fully-connected layers from Darknet-19 448 × 448 and adds a few more convolution layers for training for the object detection task. Therefore, Darknet-19 448 × 448 becomes a fully convolutional network that allows the size of the training input images to change every few iterations. This multi-scale training strategy has proven to be useful to train the neural network to adapt to different input images sizes. Overall, YOLO v2 takes advantage of this multi-scale training and prior anchor boxes to overcome many YOLO weaknesses such as localization errors and low recall problem. In our research, our marker changed its size a lot during the landing procedure; therefore, we chose YOLO v2 to train our marker detection. In our research, we replaced Darknet-19 448 × 448 with our lightDenseNet.

#### 3.2.3. Combining lightDenseNet and YOLO v2 into lightDenseYOLO

The original input image (1280 × 720 px) captured from the drone camera is resized to 320 × 320 px to match the input of lightDenseNet. In the output tensor, each 1 × 1 × 30 block stores the encoded predicted bounding box information (center coordinates (x, y), width (W), height (H), confidence score and marker class probability). By combining lightDenseNet and YOLO v2 into lightDenseYOLO, we can predict the marker at a long and close distance with a real-time speed. Table 3 shows the full description of the proposed lightDenseYOLO architecture.

### 3.3. Profile Checker v2

**Figure 8:**
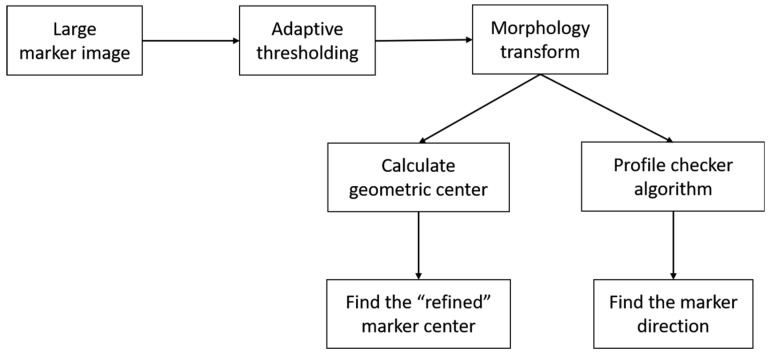
Flowchart of proposed Profile Checker v2 algorithm to find marker center and direction.

In our previous research [3], we used the Profile Checker v1 algorithm to accurately find the center of the marker from that predicted by ATM at a close distance. Even though our lightDenseYOLO predicts a much more accurate marker center than ATM (as shown in the experimental results in Section 4.2.3), there is still room for improvement. Figure 8 shows the flowchart of the proposed Profile Checker v2 to find the marker center and direction for the large predicted bounding box. The main difference we made in this new version is that we performed image transformation before using it as an input to the Profile Checker v2 algorithm.

**Figure 9:**
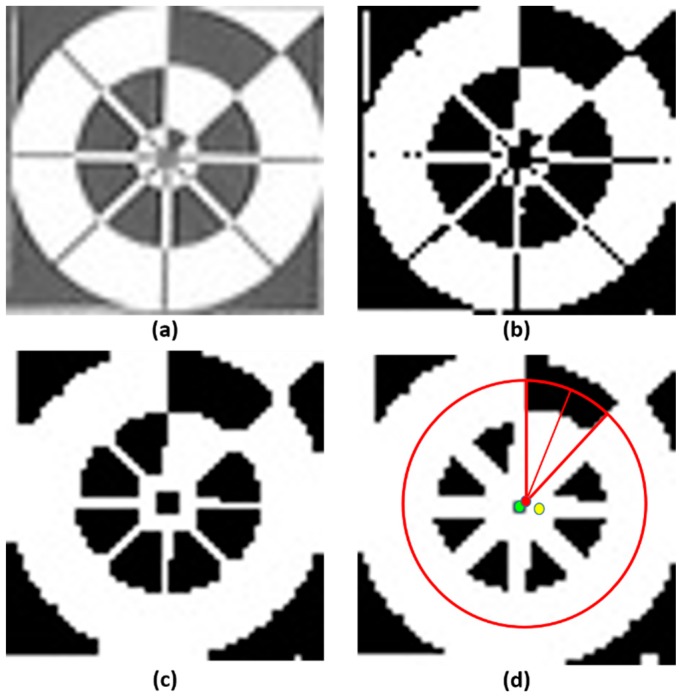
Example images of procedure of Profile Checker v2 algorithm. (**a**) Input image; (**b**) image by adaptive thresholding; (**c**) image by morphological transform; and (**d**) detected marker center and direction.

In the long-distance images, the size of the predicted marker was small; therefore, the difference between the predicted center and the ground-truth center was also small, and it was difficult to detect the marker direction in such cases. In our research, we used Profile Checker v2 algorithm to find the marker direction and the sub-optimal center of the large predicted box obtained from lightDenseYOLO. Our Profile Checker v2 algorithm starts with adaptive thresholding on the extracted marker image. The threshold value is set as the mean value of the block of an adaptive size, and we set the block size equal to a quarter of the predicted box width [46]. As shown in Figure 9b, a large amount of noise needed to be removed. To reduce the noise, we used a simple “Opening” morphology technique [47] (Figure 9c). Furthermore, dilation was performed to fill some small holes in the obtained image (Figure 9d). Next, we found the geometric center (red dot of Figure 9d). As shown in Figure 9d, compared to the predicted lightDenseYOLO center (yellow dot), the marker center predicted by Profile Checker v2 algorithm (red dot) was much closer to the ground-truth center (green dot).

**Figure 10:**
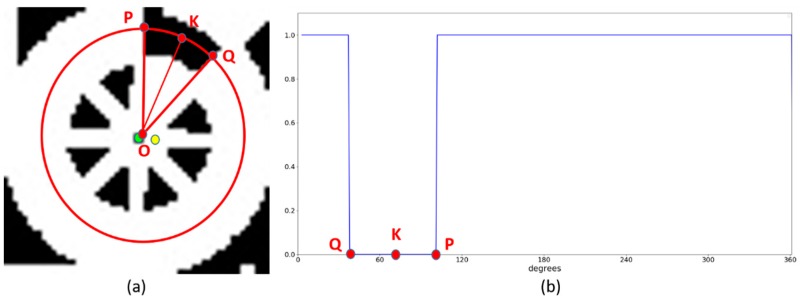
Detected center and direction of marker (**a**) using our Profile Checker v2; (**b**) Profile visualization from the red circle of Figure 10a.

To determine the direction of the marker, we used the same Profile Checker v1 method [3]. Using the obtained image (Figure 10a), we drew a circle based on the center obtained by the lightDenseYOLO, and the radius of this circle was empirically selected as 0.4× (width of marker). We refer to this circle as the profile and all its segments as sub-profiles. This profile includes all pixel values along the generated circle. With these values, we applied a threshold to create a profile with one black and one white sub-profile as shown in Figure 10b. The threshold was defined as the mean value of the maximum and minimum value of all the obtained values. A value larger than the threshold was determined as 1; otherwise, it was set to 0. From that, point “P” and “Q” were detected and the direction was estimated based on the midpoint “K” of the arc connecting “P” and “Q” with the detected center “O” of Figure 10a.

## 4. Experimental Results and Analyses

### 4.1. Experiment Hardware Platform

In this research, we used a DJI Phantom 4 drone [48] to capture landing videos. The camera settings and preprocessing steps were the same as our previous study [3]. In detail, it had a color camera with a 1/2.3-inch complementary metal-oxide-semiconductor sensor, a 94 degrees field-of-view and an f/2.8 lens. The captured videos were in mpeg-4 (MP4) format with 30 fps and had a size of 1280 × 720 px. For fast processing, the captured color image was converted to a gray image by averaging the R, G, and B pixel values. The drone’s gimbal was adjusted 90° downward so that the camera faces the ground during landing.

Previous research on CNN object detection operated on UAVs using cloud computing that sent the input image to a powerful server, and the detection results were then transferred back to the UAV for post-processing [18]. This method is not practical because it requires a stable connection between the drone and the cloud computer. In our research, we trained our lightDenseYOLO model on a desktop computer (Intel(R) Core(TM) i7-3770K CPU @3.5 GHz (4-cores) with 16 GB of RAM and NVIDIA GeForce 1070 (1920 CUDA cores) with 8-GB graphic memory [49]). Then we used the Snapdragon neural processing engine software development kit (SNPE SDK made by Qualcomm (San Diego, CA, USA)) [50] to convert and optimize our trained model on the Snapdragon 835 mobile hardware development kit [19]. The SNPE SDK enables the neural network to execute on the Snapdragon mobile platform and supports a variety of deep learning libraries such as Caffe [51], Caffe2 [52] and TensorFlow [53].

**Figure 11:**
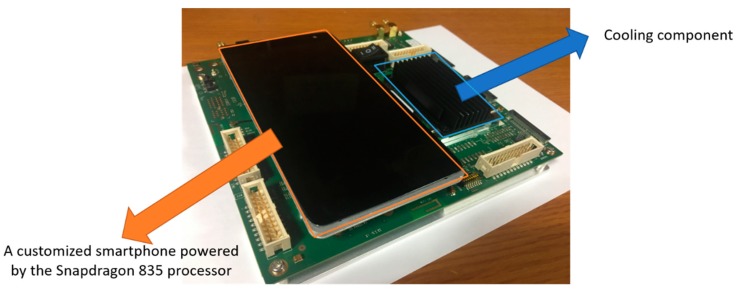
Snapdragon 835 mobile hardware development kit.

Table 4 provides a full description of the Snapdragon 835 mobile hardware development kit. The kit is a full-featured Android development platform capable of running modern CNNs. A custom mainboard is used to connect a customized smartphone with other connectivity components such as GPS, a subscriber identification module (SIM) card, and external sensors. The customized smartphone includes a 5.5-in display and a Qualcomm^®^ Snapdragon™ 835 processor (Qualcomm (San Diego, CA, USA)). The Qualcomm^®^ Snapdragon™ 835 processor carries a dual quad core Qualcomm^®^ Kryo™ 280 CPU, Qualcomm^®^ Adreno™ 540 GPU, and Qualcomm^®^ Hexagon™ DSP. The SNPE SDK converts the trained model to fully maximize the effectiveness of the Qualcomm^®^ Snapdragon™ 835 processor. We implemented our proposed CNN marker detection algorithm in this hardware kit. Our experimental hardware kit is shown in Figure 11.

### 4.2. Experiments with Self-Collected Dataset by Drone Camera

#### 4.2.1. Dongguk Drone Camera Database

**Figure 12:**
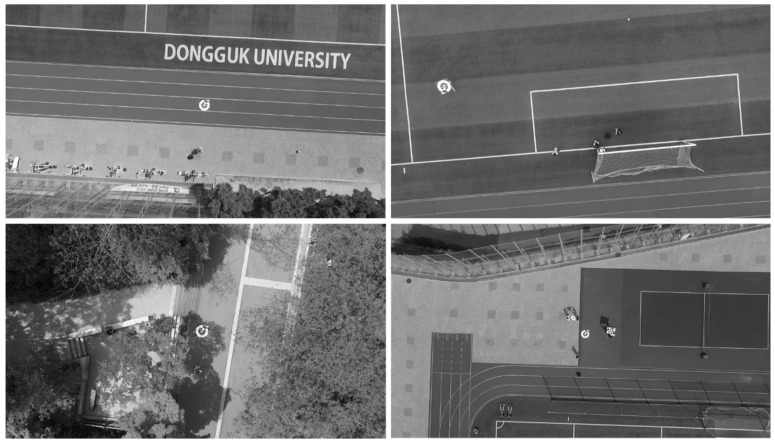
Examples of images in our DDroneC-DB2 dataset.

Our previous research successfully detected marker center and its direction at 10–15 m which is not convincing for a self-automated UAV system operated at a height of 30 m or more. In this study, we present an upgraded version of our self-constructed dataset (Dongguk Drone Camera Database (DDroneC-DB2)) which includes images from our previous dataset (DDroneC-DB1) [3] and new extracted images from videos captured from the DJI Phantom 4 drone at 50 m as shown in Figure 12. Compared to the DDroneC-DB1, our new dataset shown in Table 5 not only contained a larger number of images (10,642 images) but was also gathered in more challenging weather conditions (stronger wind velocity and long distance). For each sub-dataset, we captured videos at the morning (10 AM), afternoon (2 PM) and evening (6 PM). There are open databases acquired by drone cameras, such as Stanford Drone Dataset [54], Mini-drone Video Dataset [55], and SenseFly Dataset [56]; however, there are no open databases acquired while drones perform landing operations. Therefore, we acquired our own database for our experiments.

As explained in Section 3, we did not conduct nighttime experiments in this research because of the low-visibility of the marker in the long-distance images. Our ground-truth bounding box had the same format as the PASCAL VOC dataset [57], and it was very easy to evaluate our dataset on different CNN architectures and deep learning libraries. We made our DDroneC-DB2 dataset, trained CNN model, and algorithms public to other researchers to enable them to evaluate the performance of the marker-tracking methods [38].

To evaluate detection accuracy, we performed two-fold cross validation. For each fold, among a total of 10,642 images, we randomly chose 5231 images for training and the remainder were used for testing purposes.

#### 4.2.2. CNN Training for Marker Detection

Recent CNN object detectors such as Faster R-CNN [37] or single-shot multibox detector (SSD) [58] achieve high performance on object detection by performing fine-tuning on a well-trained backbone architecture [43,44,45] trained from scratch using the ImageNet dataset [59]. In our research, we did not train our network from scratch, but we copied the weights from the first two dense blocks of DenseNet-201 model to our first two liteDenseNet dense blocks. This DenseNet-201 model was also trained with the ImageNet dataset and yielded a performance similar to a very deep 101-layer ResNet model [42]. The weights of two additional lateral connections and additional convolutional layers of lightDenseNet were initialized by Xavier initialization [60] with zero mean and a small variance. We obtained the lightDenseYOLO model by fine-tuning the lightDenseNet model using the Darknet library [61] with two-fold cross validation. For each fold, we fine-tuned the network for 60 epochs and performed testing on the other. Throughout the training, we used a batch size of 64, a momentum of 0.9, and a decay of 0.0005. Our initial learning rate in the first 20 epochs was 10^−4^, and we reduced it by half for each of the 20 epochs until the training finished. We used the YOLO v2 data augmentation strategy with random scaling, translations, and by randomly adjusting the exposure, saturation of the image.

To make a fair comparison, we used the same two-fold cross-validation with Faster R-CNN [37], MobileNets-SSD [62], and YOLO v2 [39]. We trained the YOLO v2 marker detection model with the same hyper parameters we used for our lightDenseYOLO. We applied fine-tuning on Darknet-19 448 × 448 in the case of YOLO v2. Furthermore, we used TensorFlow object detection API [63] to train the Faster R-CNN and MobileNets-SSD models with our DDroneC-DB2. Table 6 summarizes the training parameters we used on training the marker detection model.

#### 4.2.3. Marker Detection Accuracy and Processing Time

Using DDroneC-DB2, we compared the proposed CNN marker detection (lightDenseYOLO + Profile Checker v2) with that obtained by state-of-the-art CNN object detectors such as Faster R-CNN [37], MobileNets-SSD [62], and YOLO v2 [39]. These state-of-the-art CNN object detectors were designed to predict only bounding boxes. Therefore, we adapted these schemes for marker detection using the center of the predicted bounding box as the detected center. However, these methods cannot detect the direction of a marker; therefore, we could only compare the center prediction accuracy between our lightDenseYOLO, Faster R-CNN, MobileNets-SSD, and YOLO v2 without Profile Checker v2. To have fairly compared the methods, we also applied the Profile Checker v2 algorithm in conjunction with the above methods and calculated the accuracy again. All results are the average values of two-fold cross validation.

Analysis on precision and recall at different intersection over union (IoU) thresholds has become the standard method to evaluate object detection accuracy [64,65,66]; therefore we evaluated precision and recall for different IoU thresholds. In our DDroneC-DB2, there was only one marker at each image; therefore, there was only one ground truth bounding box. If any detected bounding box had an IoU value larger than the predefined IoU threshold and the predicted class was marker, the box was considered as a true positive. Any predicted box with an IoU value smaller than the IoU threshold was considered as false positive. If there was a marker in the image but there was no detected box, it was considered as a false negative case.

At an IoU value of 0.5, Table 7 shows that the precision and recall of our lightDenseYOLO were slightly worse than those of other CNN object detectors, whereas they were better than that of YOLO v2. The reason for this is that our method was based on the YOLO detection algorithm; therefore, it obviously returned less accurate bounding boxes compared to those of the region proposal network (RPN) approaches such as Faster R-CNN. The reason why MobileNets-SSD showed better accuracy than lightDenseYOLO is that MobileNets-SSD is based on multi-scale feature maps to predict bounding boxes, whereas lightDenseYOLO is based on a single-scale feature map for fast processing. To overcome this problem, we first use lightDenseYOLO to obtain the initial prediction and then applied Profile Checker v2 to detect the precise center of the marker. Therefore, our final detected marker had a higher IoU with the ground-truth data which increased both the precision and the recall of our proposed method. As shown in Table 8, our approach (lightDenseYOLO + Profile Checker v2) performed on-par with other state-of-the-art methods such as Faster R-CNN and MobileNets-SSD. In addition, our method was better than that of lightDenseYOLO + Profile Checker v1 and YOLO v2 + Profile Checker v2.

**Figure 13:**
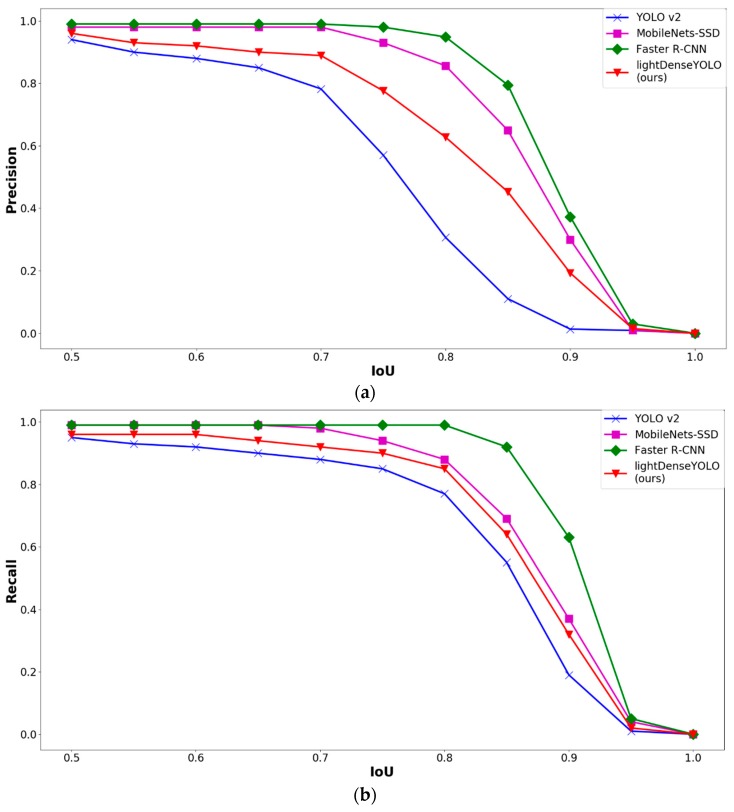
Comparative graphs of (**a**) precision and (**b**) recall by different CNN marker detectors according to intersection over union (IoU) threshold.

To investigate further, we used different IoU thresholds to see which method performed the best. Figure 13 shows the precision and recall of various CNN marker detectors at different IoU thresholds. As shown in Figure 13, both the precision and recall curves of our lightDenseYOLO were lower than those of both Faster R-CNN and MobileNets-SSD by a small margin. However, our proposed network presented superior performance against YOLO v2. The main difference between our lightDenseYOLO and YOLO v2 was the backbone architecture. Compared to Darknet-19 448 × 448, our lightDenseNet was not only deeper but also able to learn much more complex features because of the many dense connections. One of YOLO v2’s known weaknesses is having low detection accuracy on small objects. By using lightDenseNet as the feature extractor, our lightDenseYOLO overcame this weakness and detected a wide range of markers from large to small sizes with high proficiency.

**Figure 14:**
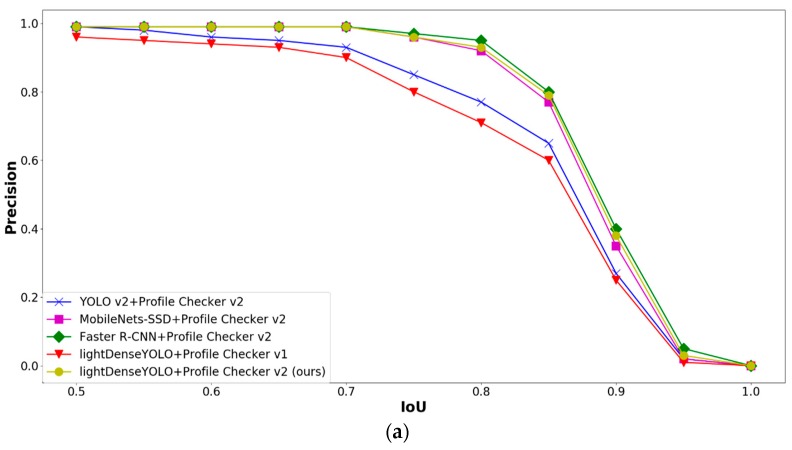

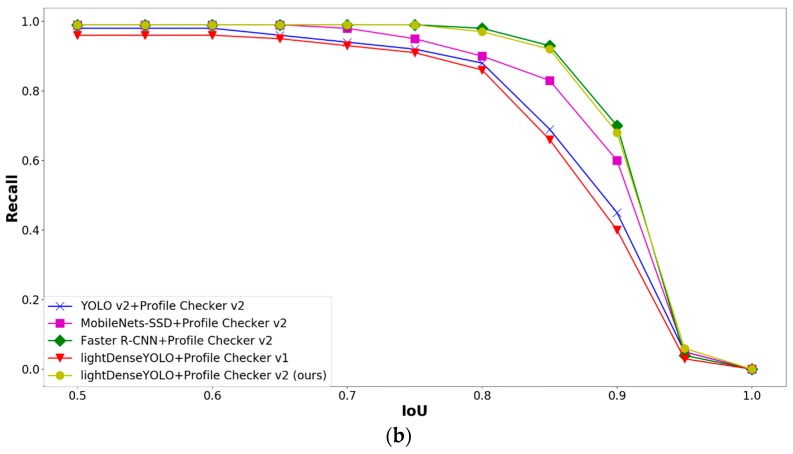
Comparative graphs of (**a**) precision and (**b**) recall by different CNN marker detectors with Profile Checker algorithms according to IoU thresholds.

**Figure 15:**
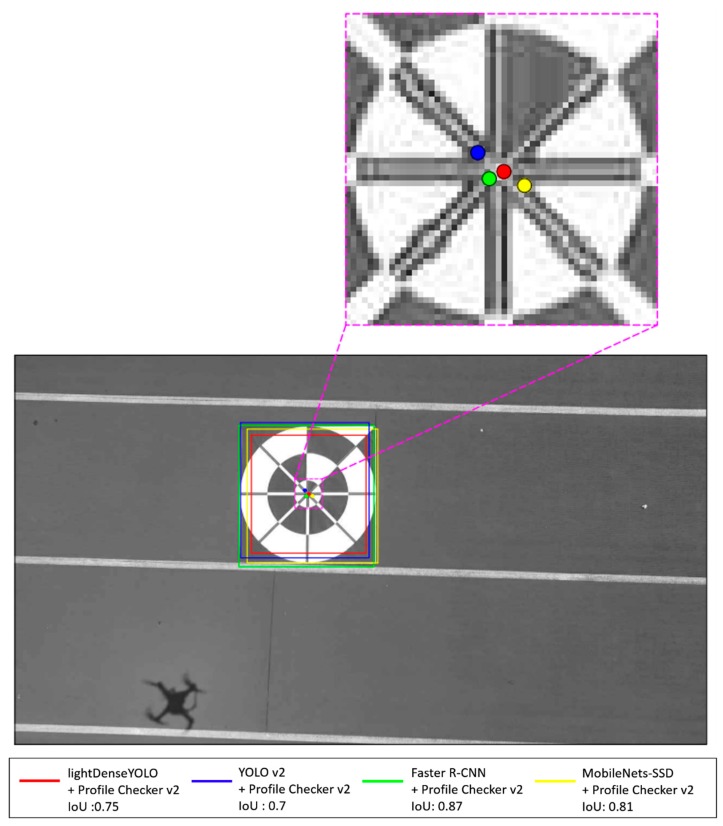
Example of detected marker at close distance image.

As shown in Figure 14, we applied Profile Checker v2 algorithms not only to our proposed network but also to other CNNs for fair comparison. It is clear that Faster R-CNN with Profile Checker v2 (green line with diamond shape) had the best precision and recall curve and our lightDenseYOLO + Profile Checker v2 (yellow line with circle shape) was the second best. Our research focused on autonomous landing; therefore, finding the marker center to guide the drone to land was the most important task. Other information such as predicted width and height of the marker was not important. In the analysis above, the first step to distinguish a predicted box as true positive or false positive was by comparing that box’s IoU value with the IoU threshold. Figure 15 shows the marker detection results of all CNN models with Profile Checker v2. All methods successfully detected the marker box, but they had different IoU values. If we set the IoU threshold value as 0.8, the predicted boxes created by our method and YOLO v2 + Profile Checker v2 were counted as false positive. Even though our method more accurately predicted the center as shown in the upper image of Figure 15, the precision and recall by our method were lower than those of the others. Therefore, measuring detection accuracy only by precision and recall cannot show the correct accuracy of our marker detection application. Therefore, we measured accuracy based on other criterion in another experiment.

**Figure 16:**
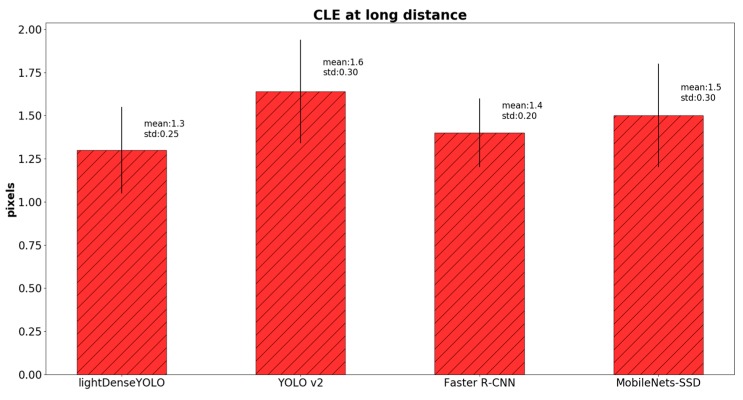
Comparison of center location error (CLE) of long-distance images of various methods.

**Figure 17:**
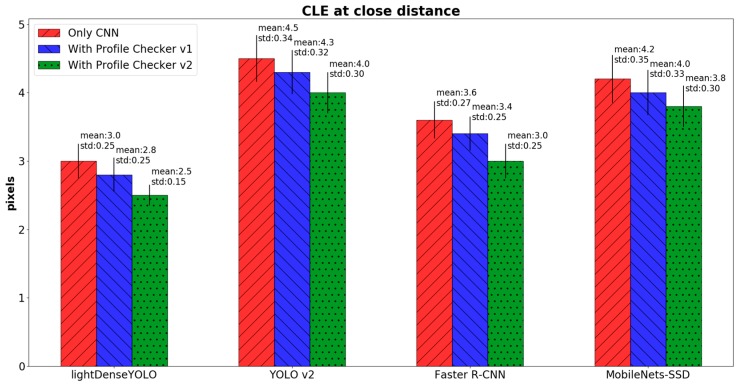
Comparison of CLE of close-distance images of various methods.

**Figure 18:**
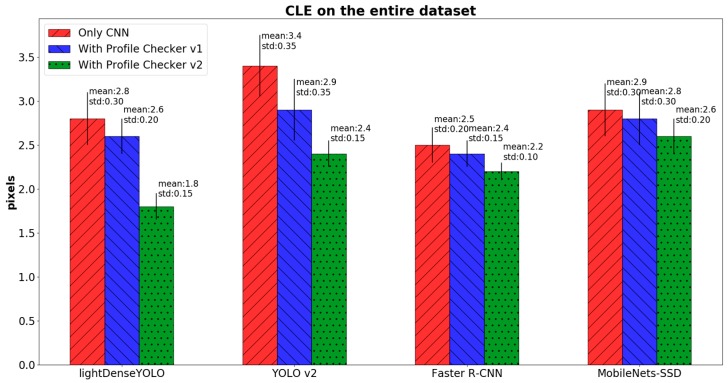
Comparison of CLE of entire DDroneC-DB2 dataset of various methods.

As the next experiment, we calculated the center location error (CLE) to identify the best method to determine the marker center. CLE is the Euclidean pixel distance between the predicted marker center and the ground-truth center shown as:

(7) $$CLE=\|O_{K}^{E}-O_{K}^{GT}\|$$

where $O_{K}^{E}$ and $O_{K}^{GT}$ are the estimated and ground-truth positions of the marker’s center, respectively. To further investigate detection accuracy, we calculated CLE in three cases: only long-distance images (Figure 16), only close-distance images (Figure 17) and the entire dataset (Figure 18). In the long-distance image case, proposed lightDenseYOLO ranked first with 1.3 px mean error, followed by Faster R-CNN, MobileNets-SSD, and YOLO v2. In addition, our proposed method (lightDenseYOLO + Profile Checker v2) outperformed the other methods as shown in Figure 17 and Figure 18.

**Figure 19:**
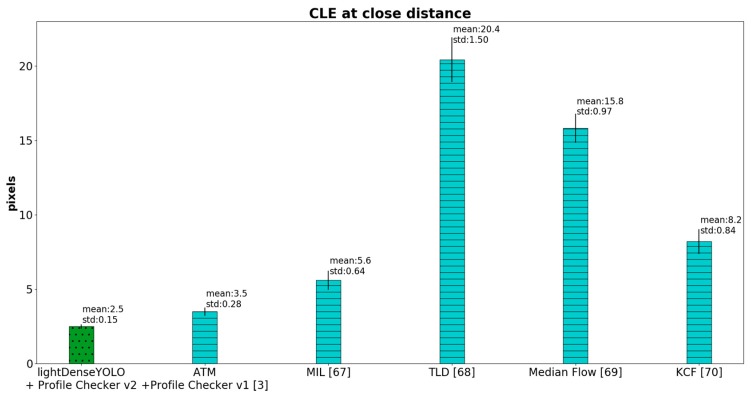
CLE comparison between our method and non-CNN marker trackers.

In Figure 19, we further compared the CLE of our CNN-based method not only with our previous remote marker-based tracking algorithm [3] but also with conventional non-CNN object trackers such as multiple instance learning (MIL) [67], tracking-learning-detection (TLD) [68], Median Flow [69], and kernelized correlation filter (KCF) [70]. Our CNN-based method showed superior detection accuracy above the other methods. These state-of-the-art object trackers [67,68,69,70] produced high CLE because they were not designed to track objects whose appearance and size keep changing in the captured image like our marker. However, our method can track a marker from a long distance even the size of the marker changes drastically.

**Figure 20:**
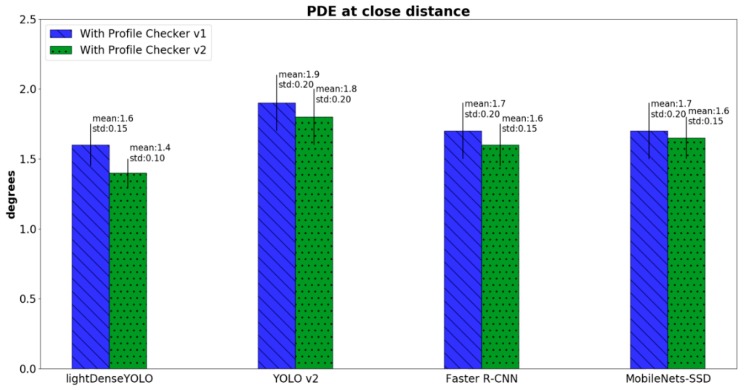
Comparison of predicted direction error (PDE) close distance images of various methods.

To compare the accuracy of predicted marker’s direction, we calculated the predicted direction error (PDE) as:

(8) $$PDE=\|D_{K}^{E}-D_{K}^{GT}\|$$

where $D_{K}^{E}$ and $D_{K}^{GT}$ are the predicted and ground-truth directions of a marker, respectively. Figure 20 shows the PDE comparison between our method and that of other CNN marker detectors. Conventional CNN detectors such as YOLO v2, Faster R-CNN, and MobileNets-SSD do not predict marker direction; therefore, we applied the Profile Checker v2 algorithm for fair comparison with our method. Our method not only had the smallest PDE, but also the smallest standard deviation as shown in Figure 20. In addition, Profile Checker v2 showed better marker-direction prediction than that of Profile Checker v1.

**Figure 21:**
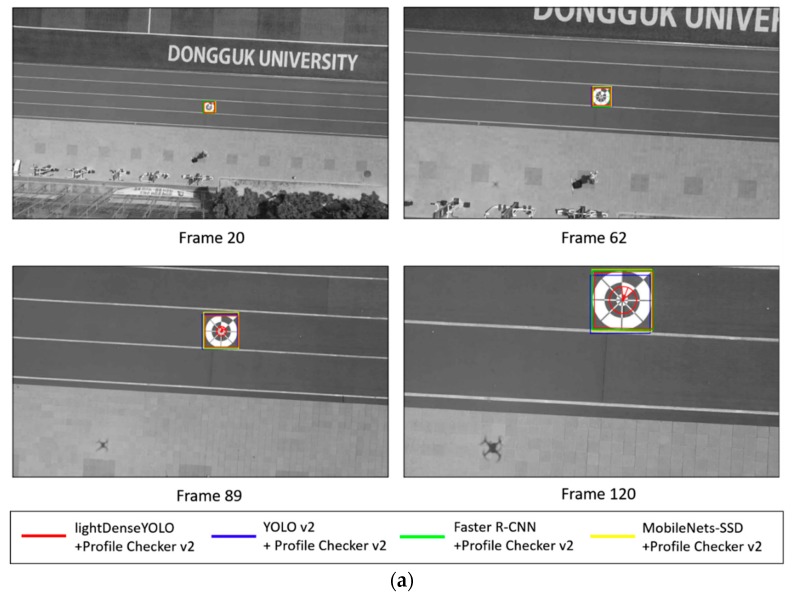

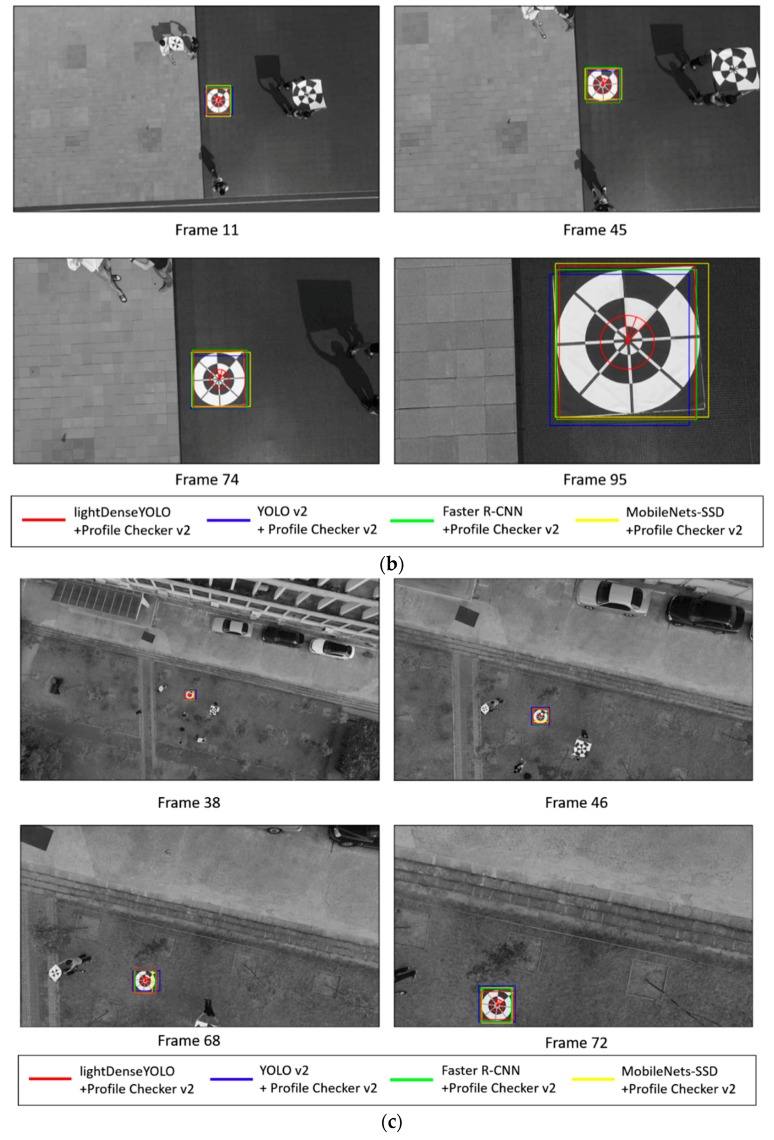
Marker detection example obtained using our method and previous methods in the (**a**) morning; (**b**) afternoon; and (**c**) evening.

Figure 21 shows the marker detection examples obtained by our method and previous methods from our DDroneC-DB2 dataset at three different time of day: morning (Figure 21a), afternoon (Figure 21b), and evening (Figure 21c). In our experiment, we used the DJI Phantom 4 remote controller with its default setting of Mode 2 (left stick controlling the throttle) and manually pushed the left stick up until the drone reached a height of 50 m. Then we let it descend using the return-to-home (RTH) function to land it safely on the ground. For simplicity, we showed the detected center and direction only by our method of lightDenseYOLO + Profile Checker v2 in red. As shown in Figure 21, the marker was successfully detected at both long and close distances in all cases, and our method showed comparatively better results than those obtained by other methods. Moreover, using the detected center obtained by our proposed method, we improved accuracy of center detection and the direction of the marker using the Profile Checker v2 algorithm. The other circular targets in Figure 21b,c are used for testing whether our method generates false positives or not. As shown in Figure 21, there were no false positive cases even with the other circular targets in the experimental images.

Table 9 shows the comparative processing speed of our method and previous methods. We measured the processing time on both a desktop computer and the Snapdragon 835 mobile hardware development kit. On a desktop computer, our lightDenseYOLO was much faster than previous methods, and our CNN architecture operated with a real-time speed of about 50 fps (20 ms per image) on the desktop computer. The Profile Checker v2 algorithm runtime was only 5 ms per image; therefore, the proposed lightDenseYOLO + Profile Checker v2 operated at about 40 fps (25 ms per image) on the desktop. In our research, we showed that our method also operates at real-time speed with the commercially available Snapdragon 835 mobile hardware development kit. We used SNPE SDK to convert the trained model into a deep learning container file so that it could be used for the Snapdragon neural processing runtime. As shown in Table 9, the processing speed of the proposed lightDenseYOLO + Profile Checker v2 was about 20 fps on the Snapdragon 835 kit, which shows that our method can be used to perform autonomous landing in a drone system powered by the Snapdragon 835 processor.

## 5. Conclusions

In this research we proposed a method for detecting a marker center and estimating the marker direction based on a light-weight CNN of lightDenseYOLO, the Profile Checker v2 algorithm, and a Kalman Filter. Our method was robust in various environmental conditions and at various distances when the marker image was captured. In addition, our method showed higher processing speed than the state-of-the-art CNN object detectors tested on desktops and with the Snapdragon 835 mobile hardware development kit. We confirmed that our method can be adopted in the embedded system of the commercially available Snapdragon 835 drone system. The errors of marker detection by our method happened when the marker was heavily occluded or moving too fast. Although our Profile Checker v2 successfully predicted a marker center and a direction in some difficult images but there are still some unsuccessful cases.

As the circle detection in our Profile Checker v2, Hough or other sophisticate circle finder can be considered. However, they take larger processing time. Because our method should be operated on the drone’s embedded system of low processing power, we used simple algorithm (the processing time for our Profile Checker v2 is just 10 ms on Snapdragon 835 embedded system as shown in Table 9). We would research more sophisticate method of circle detection such as Hough transform or others but having low processing time considering the operation on embedded system in future work. In addition, we will apply our marker-based-tracking algorithm to low-cost drones or UAVs equipped with inexpensive and low-resolution cameras to allow our works to be more easily integrated in a variety of systems. In addition, we will attempt to adopt our method to various types of images such as near infrared (NIR) or thermal images captured by drone camera and we will further research detection methods whose accuracies and processing speeds are greater than those of Faster R-CNN.

## Figures and Tables

**Table 1:** Summary of comparisons of proposed and previous studies.

Category		Type of Feature	Type of Camera	Descriptions	Strength	Weakness
Passive methods		Hand-crafted features	Multisensory fusion system with a pan-tilt unit (PTU), infrared camera, and ultra-wide-band radar, [20].	Ground-based system that first detects the unmanned aerial vehicle (UAV) in the recovery area to start tracking in the hover area and then send commands for autonomous landing.	A multiple sensor-fusion method guides UAV to land in both day and night time.	Tracking algorithm and 3D pose estimation need to be improved. Multisensory system requires complicated calibration process.
			- Ground stereo vision system with two PTUs placed on each side of a runway. - Each PTU includes a visible-light camera [21].	- Two PTUs are allocated on both sides of a runway to enlarge the baseline. - The location of the UAV is detected by the Chan-Vese model- approach and updated by an extended Kalman filter algorithm.	Ground stereo vision-based system successfully detects and tracks the UAV and shows robust detection results in real time.	Setting up two PTU ground-based systems requires extensive calibration.
			Two-infrared-camera array system with an infrared laser lamp [24].	Infrared laser lamp is fixed on the nose of the UAV for easy detection.	Infrared camera array system successfully guides the UAV to perform automatic landing in a GPS-denied environment at a distance of 1 km.	- Not practical for use in a narrow landing area. - Complicated set-up of two-camera array system on the ground is required.
Active methods	Without marker		Single down-facing visible-light camera [25].	- A local 3D elevation map of ground environment is generated using the input image from the camera sensor. - Safe landing spot is estimated by a probabilistic approach.	Without a marker, this method can help a drone find the landing spot in an emergency case.	Experiments were not conducted in various places and at different times, and the maximum height for testing was only 4–5 m.
			Infrared camera [26].	- Fixed infrared camera below the head of the UAV detects the position of four infrared lamps on a runway. - Based on prior knowledge of distance between infrared lamps, the pose parameters are calculated during the landing process.	Successfully detects infrared lamps on the ground in both day and night time at a distance of 450 m.	The series of infrared lamps required is difficult to deploy in various places.
Active methods	With marker	Hand-crafted features	Thermal camera [34,35].	Feature points are extracted from a letter-based marker enabling drone to approach closer to target and finish the landing operation.	Detect marker using thermal images and overcomes various illumination challenges.	Drone must carry a costly thermal camera.
			Visible-light camera [27,28,29,30,33].	Marker is detected by line segments or contour detectors.	Marker is detected by using only a single visible-light camera sensor.	Marker is detected only in daytime and within a limited range.
		Trained features	Visible-light camera [36].	Double-deep Q-networks solve marker detection and command the drone to reach the target simultaneously.	First approach to solve the autonomous landing problem using deep reinforcement learning.	Testing is done in an indoor environment, and there is a gap between indoor and outdoor environments.
			Visible-light camera (proposed method).	- Uses lightweight lightDenseYOLO convolutional neural network (CNN) marker detector to roughly predict marker location. - Enhanced detection of marker center and direction is performed by Profile Checker v2 algorithm.	- Requires only a single visible-light camera. - Detects marker center and direction at very long distance at a fast speed.	An embedded system which can support deep learning is required to operate marker detection in real time.

**Table 2:** Differences of characteristics of YOLO and YOLO v2 (unit: px).

Characteristic		YOLO	YOLO v2
Feature Extractor		Darknet	Darknet-19 448 × 448
Input size	Training from scratch using ImageNet dataset	224 × 224	448 × 448
	Training by fine-tuning using Pascal VOC or MS COCO dataset	448 × 448	448 × 448
	Testing	448 × 448	448 × 448

**Table 3:** Architecture of lightDenseYOLO for marker detection. Each conv layer is the sequence of batch normalization (BN), rectified linear unit (ReLU), and convolution layer. s1 and s2 present stride by 1 and 2 px, respectively.

Layer		Input Size	Output Size
Input		320 × 320 × 3	320 × 320 × 3
7 × 7 conv, s2		320 × 320 × 3	160 × 160 × 64
2 × 2 pooling, s2		160 × 160 × 64	80 × 80 × 64
Dense block 1	$\left[\begin{array}{c} 1\;\times \;1\;conv,\;s1\\ 3\;\times \;3\;conv,\;s1 \end{array}\right]\times \;6$	80 × 80 × 64	80 × 80 × 256
Transition layer	$\left[\begin{array}{c} 1\;\times \;1\;conv,\;s1\\ 2\;\times \;2\;pooling,\;s2 \end{array}\right]$	80 × 80 × 256	40 × 40 × 128
Dense block 2	$\left[\begin{array}{c} 1\;\times \;1\;conv,\;s1\\ 3\;\times \;3\;conv,\;s1 \end{array}\right]\times 12$	40 × 40 × 128	40 × 40 × 512
Transition layer	$\left[\begin{array}{c} 1\;\times \;1\;conv,\;s1\\ 2\;\times \;2\;pooling,\;s2 \end{array}\right]$	40 × 40 × 512	20 × 20 × 256
Reshape		40 × 40 × 320	20 × 20 × 1280
Bottleneck layer	$\left[\begin{array}{c} 1\;\times \;1\;conv,\;s1\\ 3\;\times \;3\;conv,\;s1 \end{array}\right]$	20 × 20 × 1280	20 × 20 × 32
Reshape		80 × 80 × 128	20 × 20 × 2048
Bottleneck layer	$\left[\begin{array}{c} 1\;\times \;1\;conv,\;s1\\ 3\;\times \;3\;conv,\;s1 \end{array}\right]$	20 × 20 × 2048	20 × 20 × 32
Concatenation		20 × 20 × 32 20 × 20 × 32 20 × 20 × 256	20 × 20 × 320
[3×3 conv, s1] × 3		20 × 20 × 320	20 × 20 × 30

**Table 4:** Description of Snapdragon 835 mobile hardware development kit.

Components	Specifications
Central Processing Unit (CPU)	Qualcomm^®^ Kryo™ 280 (dual-quad core, 64-bit ARM V8 compliant processors, 2.2 GHz and 1.9 GHz clusters)
Graphics Processing Unit (GPU)	Qualcomm^®^ Adreno™ 540
Digital Processing Unit (DSP)	Qualcomm^®^ Hexagon™ DSP with Hexagon vector extensions
RAM	4 GB
Storage	128 GB
Operating System	Android 7.0 “Nougat”

**Table 5:** Description of DDroneC-DB2.

Sub-Dataset		Number of Images	Condition	Description
Morning	Far	3088	Humidity: 44.7% Wind speed: 5.2 m/s Temperature: 15.2 °C, autumn, sunny Illuminance:1800 lux	Landing speed: 5.5 m/s Auto mode of camera shutter speed (8~1/8000 s) and ISO (100~3200)
	Close	641		
	Close (from DdroneC-DB1 [3])	425	Humidity: 41.5% Wind speed: 1.4 m/s Temperature: 8.6 °C, spring, sunny Illuminance: 1900 lux	Landing speed: 4 m/sAuto mode of camera shutter speed(8~1/8000 s) and ISO (100~3200)
Afternoon	Far	2140	Humidity: 82.1% Wind speed: 6.5 m/s Temperature: 28 °C, summer, sunny Illuminance:2250 lux	Landing speed: 7 m/s Auto mode of camera shutter speed (8~1/8000 s) and ISO (100~3200)
	Close	352		
	Close (from DdroneC-DB1 [3])	148	Humidity: 73.8% Wind speed: 2 m/s Temperature: −2.5 °C, winter, cloudy Illuminance: 1200 lux	Landing speed: 6 m/s Auto mode of camera shutter speed (8~1/8000 s) and ISO (100~3200)
Evening	Far	3238	Humidity: 31.5% Wind speed: 7.2 m/s Temperature: 6.9 °C, autumn, foggy Illuminance: 650 lux	Landing speed: 6 m/s Auto mode of camera shutter speed (8~1/8000 s) and ISO (100~3200)
	Close	326		
	Close (from DdroneC-DB1 [3])	284	Humidity: 38.4% Wind speed: 3.5 m/s Temperature: 3.5 °C, winter, windy Illuminance: 500 lux	Landing speed: 4 m/s Auto mode of camera shutter speed (8~1/8000 s) and ISO (100~3200)

**Table 6:** Summary of training hyper parameters used on different models for DDroneC-DB2 dataset.

	lightDenseYOLO (Ours)	YOLO v2	Faster R-CNN	MobileNets-SSD
Input size (unit: px)	Multi-scale training (from 128 × 128 to 640 × 640)	Multi-scale training (from 128 × 128 to 640 × 640)	320 × 320	320 × 320
Number of epochs	60	60	60	60
Batch size	64	64	64	64
Initial learning rate	0.0001	0.0001	0.0001	0.004
Momentum	0.9	0.9	0.9	0.9
Decay	0.0005	0.0005	0.0005	0.9
Backbone architecture	lightDenseNet	Darknet-19 448 × 448	VGG 16	MobileNets

**Table 7:** Precision (P) and recall (R) at IoU = 0.5 of different CNN marker detectors.

	Morning				Afternoon				Evening				Entire Dataset					
	Far		Close		Far		Close		Far		Close		Far		Close		Far + Close	
	P	R	P	R	P	R	P	R	P	R	P	R	P	R	P	R	P	R
lightDenseYOLO	0.96	0.95	0.96	0.96	0.94	0.96	0.95	0.95	0.95	0.96	0.97	0.96	0.95	0.96	0.96	0.96	0.96	0.96
YOLO v2	0.95	0.95	0.96	0.95	0.92	0.94	0.93	0.95	0.94	0.93	0.95	0.96	0.94	0.94	0.95	0.95	0.94	0.95
Faster R-CNN	0.99	0.99	0.99	0.99	0.99	0.98	0.98	0.99	0.99	0.99	0.99	0.98	0.99	0.99	0.99	0.99	0.99	0.99
MobileNets-SSD	0.98	0.98	0.99	0.98	0.97	0.96	0.97	0.98	0.98	0.97	0.97	0.99	0.98	0.97	0.98	0.98	0.98	0.98

**Table 8:** Precision (P) and recall (R) at IoU = 0.5 of different CNN marker detectors with Profile Checker algorithms.

	Morning				Afternoon				Evening				Entire Dataset					
	Far		Close		Far		Close		Far		close		Far		Close		Far +close	
	P	R	P	R	P	R	P	R	P	R	P	R	P	R	P	R	P	R
lightDenseYOLO +Profile Checker v1	0.97	0.96	0.96	0.95	0.96	0.97	0.96	0.98	0.95	0.96	0.96	0.96	0.96	0.96	0.96	0.96	0.96	0.96
lightDenseYOLO +Profile Checker v2	0.99	0.99	0.98	0.99	0.98	0.98	0.99	0.98	0.98	0.99	0.99	0.99	0.98	0.99	0.99	0.99	0.99	0.99
YOLO v2 + Profile Checker v2	0.98	0.97	0.99	0.99	0.98	0.99	0.99	0.98	0.98	0.98	0.99	0.99	0.98	0.98	0.99	0.99	0.99	0.98
Faster R-CNN + Profile Checker v2	0.99	0.99	0.99	0.99	0.99	0.99	0.99	0.99	0.99	0.99	0.99	0.99	0.99	0.99	0.99	0.99	0.99	0.99
MobileNets-SSD + Profile Checker v2	0.99	0.99	0.99	0.99	0.99	0.99	0.99	0.99	0.99	0.99	0.99	0.99	0.99	0.99	0.99	0.99	0.99	0.99

**Table 9:** Comparisons of average processing speed of proposed method with those of other CNN marker detectors (fps).

	Desktop Computer	Snapdragon 835 kit
lightDenseYOLO	~50	~25
YOLO v2	~33	~9.2
Faster R-CNN	~5	~2.5
MobileNets-SSD	~12.5	~7.14
lightDenseYOLO + Profile Checker v1	~40	~20.83
lightDenseYOLO + Profile Checker v2	~40	~20
YOLO v2 + Profile Checker v2	~28.6	~7.7
Faster R-CNN + Profile Checker v2	~4.87	~2
MobileNets-SSD + Profile Checker v2	~11.8	~6.75

## References

[1] The First Autonomous Drone Delivery Network will fly above Switzerland Starting next Month. https://www.theverge.com/2017/9/20/16325084/matternet-autonomous-drone-network-switzerland.

[2] Amazon Prime Air. https://www.amazon.com/Amazon-Prime-Air/b?ie=UTF8&node=8037720011.

[3] Nguyen P.H., Kim K.W., Lee Y.W., Park K.R. (2017). Remote marker-based tracking for UAV landing using visible-light camera sensor. Sensors.

[4] Schmidhuber J. (2015). Deep learning in neural networks: An overview. Neural Netw..

[5] Amer K., Samy M., ElHakim R., Shaker M., ElHelw M. Convolutional neural network-based deep urban signatures with application to drone localization. Proceedings of the IEEE International Conference on Computer Vision Workshops.

[6] Chen Y., Aggarwal P., Choi J., Kuo C.-C.J. (2017). A deep learning approach to drone monitoring. arXiv.

[7] Saqib M., Khan S.D., Sharma N., Blumenstein M. A study on detecting drones using deep convolutional neural networks. Proceedings of the 14th IEEE International Conference on Advanced Video and Signal Based Surveillance.

[8] Kim B.K., Kang H.-S., Park S.-O. (2017). Drone classification using convolutional neural networks with merged doppler images. IEEE Geosci. Remote Sens. Lett..

[9] Tzelepi M., Tefas A. Human crowd detection for drone flight safety using convolutional neural networks. Proceedings of the 25th European Signal Processing Conference.

[10] Burman P. (2016). Quadcopter Stabilization with Neural Network. Master‘s Thesis.

[11] Greenwood D. The application of neural networks to drone control. Proceedings of the International Telemetering Conference.

[12] Kim D.K., Chen T. (2015). Deep neural network for real-time autonomous indoor navigation. arXiv.

[13] Andersson O., Wzorek M., Doherty P. Deep learning quadcopter control via risk-aware active learning. Proceedings of the Thirty-First AAAI Conference on Artificial Intelligence.

[14] Gandhi D., Pinto L., Gupta A. Learning to fly by crashing. Proceedings of the IEEE/RSJ International Conference on Intelligent Robots and Systems.

[15] Giusti A., Guzzi J., Cireşan D.C., He F.-L., Rodríguez J.P., Fontana F., Faessler M., Forster C., Schmidhuber J., Caro G.D. (2016). A machine learning approach to visual perception of forest trails for mobile robots. IEEE Robot. Autom. Lett..

[16] Smolyanskiy N., Kamenev A., Smith J., Birchfield S. (2017). Toward low-flying autonomous MAV trail navigation using deep neural networks for environmental awareness. arXiv.

[17] Radovic M., Adarkwa O., Wang Q. (2017). Object recognition in aerial images using convolutional neural networks. J. Imaging.

[18] Lee J., Wang J., Crandall D., Šabanović S., Fox G. Real-time, cloud-based object detection for unmanned aerial vehicles. Proceedings of the First IEEE International Conference on Robotic Computing.

[19] Snapdragon 835 Mobile Hardware Development Kit. https://developer.qualcomm.com/hardware/snapdragon-835-hdk.

[20] Zhou D., Zhong Z., Zhang D., Shen L., Yan C. Autonomous landing of a helicopter UAV with a ground-based multisensory fusion system. Proceedings of the Seventh International Conference on Machine Vision.

[21] Tang D., Hu T., Shen L., Zhang D., Kong W., Low K.H. (2016). Ground stereo vision-based navigation for autonomous take-off and landing of UAVs: A Chan-Vese model approach. Int. J. Adv. Robot. Syst..

[22] Chan T.F., Vese L.A. (2001). Active contours without edges. IEEE Trans. Image Process..

[23] Kalman R.E. (1960). A new approach to linear filtering and prediction problems. J. Basic Eng..

[24] Yang T., Li G., Li J., Zhang Y., Zhang X., Zhang Z., Li Z. (2016). A ground-based near infrared camera array system for UAV auto-landing in GPS-denied environment. Sensors.

[25] Forster C., Faessler M., Fontana F., Werlberger M., Scaramuzza D. Continuous on-board monocular-vision-based elevation mapping applied to autonomous landing of micro aerial vehicles. Proceedings of the IEEE International Conference on Robotics and Automation.

[26] Gui Y., Guo P., Zhang H., Lei Z., Zhou X., Du J., Yu Q. (2013). Airborne vision-based navigation method for UAV accuracy landing using infrared lamps. J. Intell. Robot. Syst..

[27] Lin S., Garratt M.A., Lambert A.J. (2017). Monocular vision-based real-time target recognition and tracking for autonomously landing an UAV in a cluttered shipboard environment. Auton. Robots.

[28] Lange S., Sünderhauf N., Protzel P. A vision based onboard approach for landing and position control of an autonomous multirotor UAV in GPS-denied environments. Proceedings of the IEEE International Conference on Advanced Robotics.

[29] Polvara R., Sharma S., Wan J., Manning A., Sutton R. Towards autonomous landing on a moving vessel through fiducial markers. Proceedings of the European Conference on Mobile Robotics.

[30] Falanga D., Zanchettin A., Simovic A., Delmerico J., Scaramuzza D. Vision-based autonomous quadrotor landing on a moving platform. Proceedings of the IEEE International Symposium on Safety, Security and Rescue Robotics.

[31] AprilTag. https://april.eecs.umich.edu/software/apriltag.html.

[32] Wang J., Olson E. AprilTag 2: Efficient and robust fiducial detection. Proceedings of the IEEE/RSJ International Conference on Intelligent Robots and Systems.

[33] Araar O., Aouf N., Vitanov I. (2017). Vision based autonomous landing of multirotor UAV on moving platform. J. Intell. Robot. Syst..

[34] Xu G., Zhang Y., Ji S., Cheng Y., Tian Y. (2009). Research on computer vision-based for UAV autonomous landing on a ship. Pattern Recognit. Lett..

[35] Xu G., Qi X., Zeng Q., Tian Y., Guo R., Wang B. (2013). Use of land’s cooperative object to estimate UAV’s pose for autonomous landing. Chin. J. Aeronaut..

[36] Polvara R., Patacchiola M., Sharma S., Wan J., Manning A., Sutton R., Cangelosi A. (2017). Autonomous quadrotor landing using deep reinforcement learning. arXiv.

[37] Ren S., He K., Girshick R., Sun J. (2017). Faster R-CNN: Towards real-time object detection with region proposal networks. IEEE Trans. Pattern Anal. Mach. Intell..

[38] Dongguk Drone Camera Database (DDroneC–DB2) & lightDenseYOLO. http://dm.dgu.edu/link.html.

[39] Redmon J., Farhadi A. YOLO9000: Better, faster, stronger. Proceedings of the IEEE Conference on Computer Vision and Pattern Recognition.

[40] Welch G., Bishop G. An introduction to the Kalman filter. Proceedings of the Special Interest Group on GRAPHics and Interactive Techniques.

[41] Redmon J., Divvala S., Girshick R., Farhadi A. You only look once: Unified, real-time object detection. Proceedings of the IEEE Conference on Computer Vision and Pattern Recognition.

[42] Huang G., Liu Z., van der Maaten L., Weinberger K.Q. Densely connected convolutional networks. Proceedings of the IEEE Conference on Computer Vision and Pattern Recognition.

[43] Krizhevsky A., Sutskever I., Hinton G.E. ImageNet classification with deep convolutional neural networks. Proceedings of the International Conference on Neural Information Processing Systems 25.

[44] Simonyan K., Zisserman A. Very deep convolutional networks for large-scale image recognition. Proceedings of the 3rd International Conference on Learning Representations.

[45] He K., Zhang X., Ren S., Sun J. Deep residual learning for image recognition. Proceedings of the IEEE International Conference on Computer Vision and Pattern Recognition.

[46] OpenCV Image Thresholding. https://docs.opencv.org/3.4.0/d7/d4d/tutorial_py_thresholding.html.

[47] OpenCV Morphological Transformations. https://docs.opencv.org/trunk/d9/d61/tutorial_py_morphological_ops.html.

[48] Phantom 4. https://www.dji.com/phantom-4.

[49] GeForce GTX 1070. https://www.geforce.com/hardware/desktop-gpus/geforce-gtx-1070/specifications.

[50] Qualcomm Snapdragon Neural Processing Engine for AI. https://developer.qualcomm.com/software/snapdragon-neural-processing-engine-ai.

[51] Jia Y., Shelhamer E., Donahue J., Karayev S., Long J., Girshick R., Guadarrama S., Darrell T. (2014). Caffe: Convolutional architecture for fast feature embedding. arXiv.

[52] Caffe2. http://caffe2.ai/.

[53] TensorFlow. https://www.tensorflow.org/.

[54] Stanford Drone Dataset. http://cvgl.stanford.edu/projects/uav_data/.

[55] Mini–Drone Video Dataset. http://mmspg.epfl.ch/mini-drone.

[56] SenseFly Dataset. https://www.sensefly.com/education/datasets/.

[57] Everingham M., Eslami S.M.A., van Gool L., Williams C.K.I., Winn J., Zisserman A. (2015). The Pascal visual object classes challenge: A retrospective. Int. J. Comput. Vis..

[58] Liu W., Anguelov D., Erhan D., Szegedy C., Reed S., Fu C.-Y., Berg A.C. SSD: Single shot multibox detector. Proceedings of the 14th European Conference on Computer Vision.

[59] Deng J., Dong W., Socher R., Li L.-J., Li K., Fei-Fei L. ImageNet: A large-scale hierarchical image database. Proceedings of the IEEE Conference on Computer Vision and Pattern Recognition.

[60] Glorot X., Bengio Y. Understanding the difficulty of training deep feedforward neural networks. Proceedings of the 13th International Conference on Artificial Intelligence and Statistics.

[61] Darknet: Open Source Neural Networks in C. https://pjreddie.com/darknet/.

[62] Howard A.G., Zhu M., Chen B., Kalenichenko D., Wang W., Weyand T., Andreetto M., Adam H. (2017). MobileNets: Efficient convolutional neural networks for mobile vision applications. arXiv.

[63] Huang J., Rathod V., Sun C., Zhu M., Korattikara A., Fathi A., Fischer I., Wojna Z., Song Y., Guadarrama S. Speed/accuracy trade-offs for modern convolutional object detectors. Proceedings of the IEEE Conference on Computer Vision and Pattern Recognition.

[64] Hosang J., Benenson R., Schiele B. (2014). How good are detection proposals, really?. arXiv.

[65] Hosang J., Benenson R., Dollár P., Schiele B. (2016). What makes for effective detection proposals?. IEEE Trans. Pattern Anal. Mach. Intell..

[66] Chavali N., Agrawal H., Mahendru A., Batra D. Object-proposal evaluation protocol is ‘gameable’. Proceedings of the IEEE Conference on Computer Vision and Pattern Recognition.

[67] Babenko B., Yang M.H., Belongie S. Visual tracking with online multiple instance learning. Proceedings of the IEEE Conference on Computer Vision and Pattern Recognition.

[68] Kalal Z., Mikolajczyk K., Matas J. (2012). Tracking-learning-detection. IEEE Trans. Pattern Anal. Mach. Intell..

[69] Kalal Z., Mikolajczyk K., Matas J. Forward–backward error: Automatic detection of tracking failures. Proceedings of the International Conference on Pattern Recognition.

[70] Henriques J.F., Caseiro R., Martins P., Batista J. (2015). High–speed tracking with kernelized correlation filters. IEEE Trans. Pattern Anal. Mach. Intell..

